# Hematopoietic stem/progenitor cell transplantation recovers immune defects and prevents lymphomas in Atm-deficient mice

**DOI:** 10.1186/s40164-024-00544-0

**Published:** 2024-08-06

**Authors:** Bruna Sabino Pinho de Oliveira, Alessandro Giovinazzo, Sabrina Putti, Matilde Merolle, Tiziana Orsini, Giuseppe D. Tocchini-Valentini, Christophe Lancrin, Fabio Naro, Manuela Pellegrini

**Affiliations:** 1 Institute of Biochemistry and Cell Biology (IBBC- CNR), Via Ercole Ramarini, 32 Monterotondo Scalo, 00015 Rome, Italy; 2 Department of Anatomical, Histological, Forensic and Orthopaedic Sciences, Sapienza University of Rome, 00161 Rome, Italy; 3 European Mouse Mutant Archive (EMMA), INFRAFRONTIER-IMPC, Mouse Clinic-CNR, Monterotondo Scalo, 00015 Rome, Italy; 4 Epigenetics and Neurobiology Unit, European Molecular Biology Laboratory (EMBL), Monterotondo Scalo, 00015 Rome, Italy

**Keywords:** ATM, HSPCT, Lymphomas, T and B cells, Genomic stability

## Abstract

**Background:**

Ataxia-telangiectasia (A-T) is a rare autosomal recessive multi-system and life-shortening disease, characterized by progressive cerebellar neurodegeneration, immunodeficiency, radiation sensitivity and cancer predisposition, with high incidence of leukemia and lymphoma. A-T is caused by mutations in the gene encoding for ATM protein that has a major role in maintaining the integrity of the genome. Because there are no cures for A-T, we aimed to tackle immunodeficiency and prevent cancer onset/progression by transplantation therapy.

**Methods:**

Enriched hematopoietic stem/progenitor cells (HSPCs), collected from bone marrow of wild-type mice, were transplanted in the caudal vein of 1 month old conditioned *Atm*^*−/−*^ mice.

**Results:**

Genomic analyses showed that transplanted Atm positive cells were found in lymphoid organs. B cells isolated from spleen of transplanted mice were able to undergo class switching recombination. Thymocytes were capable to correctly differentiate and consequently an increase of helper T cells and TCRβ^hi^ expressing cells was observed. Protein analysis of isolated T and B cells from transplanted mice, revealed that they expressed Atm and responded to DNA damage by initiating an Atm-dependent phosphorylation cascade. Indeed, aberrant metaphases were reduced in transplanted Atm-deficient mice. Six months after transplantation, *Atm*^*−/−*^ mice showed signs of aging, but they maintained the rescue of T cells maturation, showed DNA damage response, and prevented thymoma.

**Conclusion:**

We can conclude that wild-type enriched HSPCs transplantation into young Atm-deficient mice can ameliorate A-T hematopoietic phenotypes and prevent tumor of hematopoietic origin.

**Supplementary Information:**

The online version contains supplementary material available at 10.1186/s40164-024-00544-0.

## To the Editor,

Ataxia-Telangiectasia (A-T) is a rare recessive autosomal disorder, commonly referred as genome instability syndrome and as primary immunodeficiency (PID). A-T patients suffer of neurodegeneration, aging, sterility, radiosensitivity and cancer predisposition. The cause of the disease are mutations in the *ATM* (ataxia telangiectasia mutated) gene that encodes for a serine/threonine kinase of the phosphatidylinositol-3-kinase related kinase (PI3KK) family, majorly involved in cell cycle regulation and DNA repair mechanisms [1]. Malignancies of hematological origin, specifically affecting B and T cell lineages, are frequent in A-T patients, with a high incidence of 21.7% by the age of 15 years [2–4]. The most common forms are Acute Lymphocytic Leukemias (ALL), non-Hodgkin’s and Hodgkin’s lymphomas. In older patients are observed Chronic T-Cell Leukemias (CCL) and solid tumors, including gastric, colon, pancreas, breast, liver, esophageal and basal cell carcinomas, ovarian dysgerminoma and uterine leiomyoma [3].

Currently, there is no cure for the disease and A-T patients die before their forties while treatments are only symptomatic and supportive [5–7]. Bone marrow heterologous transplantation is one of the therapeutic options that could tackle immunodeficiency, as it is for other PIDs, but due to the extreme radiosensitivity of the patients and non-myeloablative conditioning side effects, its use has been restricted and the outcomes are highly unpredictable [8]. Gene therapy strategies could be then a favourable alternative to the allo-transplantation, since correcting patients’ own cells will allow autologous transplantation, avoiding most of side effects such as graft vs host disease, tissue rejection, infections, and the need of Human Leukocyte Antigen (HLA)-identical related donors [9]. In this scenario, it becomes urgent to identify the critical hematopoietic cell population to be eventually engineered with *Atm* and transplanted to counteract immunodeficiency and hematopoietic cancer predisposition of A-T disease.

Previous works demonstrated the feasibility of bone marrow transplantation in Atm-deficient mice and the amelioration of the phenotype [10–13] and we have already proved that global Atm reactivation can revert A-T in transgenic mouse models [14]. In this study, we expand on prior research describing the transplantation and engraftment of wild-type hematopoietic stem/progenitor cells (HSPCs) into *Atm*^*−/−*^ mice as well as their effect on immunological markers, genomic stability and DNA damage response. Using magnetic beads, we selected Lineage negative and c-Kit positive (Lin^−^c-Kit^+^, LK) cells from bone marrow, that are known to be hematopoietic stem and progenitor cells [15] and so B and T cell progenitors. The final enriched LK population was around 3% of harvested cells and c-Kit^+^ cells both Sca-1^+^ and Sca-1^−^ reached around 80% of purification (Suppl. Figure 1a). This population was maintained 24 h after culture with the significant increase of c-Kit^+^Sca-1^+^ subpopulation (Suppl. Figure 1a), already known to be capable of reconstituting hematopoiesis in lethally irradiate wild-type mice [15]. A further characterization revealed that also the proportion of CD150^+^ hematopoietic stem cells (HSCs) was maintained, whereas a significant increase was observed in the CD48^+^ hematopoietic progenitor cells (HPCs) subset (Suppl. Figure 1b).

Before transplantation, we verified the efficacy of the conditioning approach on *Atm*^*−/−*^ mice [11–13], and extremely decreased T cells percentage was observed in peripheral blood for up to 7 weeks (Suppl. Figure 1c).

Then, we transplanted 3 to 5 million LK-enriched cells from wild-type male donors into conditioned *Atm*^*−/−*^ female recipients and analyzed the LK-transplanted female mice (*Atm*^*LKT*^) as shown in the flow chart reported in Suppl. Figure 1d.

**Fig. 1:**
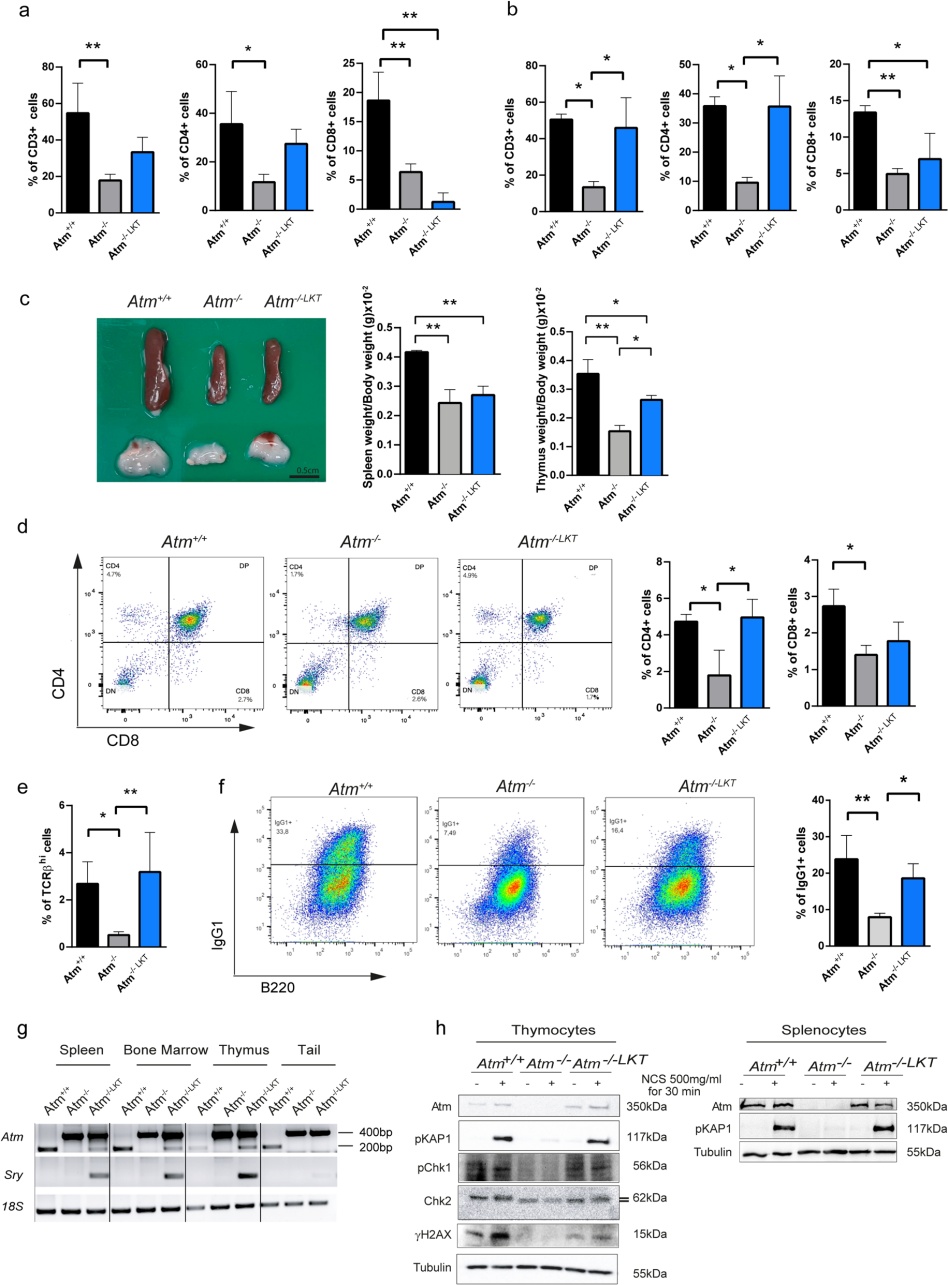
Restoration of T cells in *Atm*^*−*/^^*−*^ systemic blood and thymus, and B-cells in spleen after *Atm*^*+*/*+*^ LK cells transplantation. **a** Histograms of total, helper and cytotoxic T cells (CD3, CD4, CD8) in peripheral blood 4 weeks after transplantation. **b** Histograms of total, helper and cytotoxic T cells (CD3, CD4, CD8) in peripheral blood 7 weeks after transplantation. **c** Thymus and spleen picture and relative weight bar charts. The ratio between organ weight (g) and body weight (g) is reported. **d** Representative flow cytometry panels of CD4 and CD8 T cells and relative histogram of mean values of CD4 and CD8 single positive T cells are shown. **e** Histogram of mean values of TCRβ^hi^ expressing cells analyzed by flow cytometry. **f** Representative flow cytometry dot-plot of B cells class switching cultured in LPS and IL4 for 96 h and relative histogram of mean values of IgG1 expressing cells. **g** Representative genomic PCR analysis of mouse tissues collected 7 weeks after LKT. The *Atm* 200 bp band identifies the wild-type sequence of *Atm* gene, whereas the *Atm* 400 bp band identifies the knockout sequence of *Atm* gene. The *Sry* 100 bp band identifies male cells in the female background and *18 s* *rRNA* gene is used as a housekeeping sequence. **h** Western blot analysis of Atm expression and DNA damage response in isolated thymocytes and splenocytes. Neocarzinostatin (NCS) was used as DNA damage inducer. Phosphorylation of KAP1, Chk1, Chk2 (upper band) and H2AX (γH2AX) indicate DNA damage response. Tubulin was used as loading control. N = 3 mice of each group. *P ≤ 0.05 and ** P ≤ 0.01

We found substantial improvements in CD3 positive T cells and CD4 single-positive helper T cells in transplanted mice compared to *Atm*^*−/−*^ animals in peripheral blood 4 and 7 weeks after transplantation (Fig. 1a,b). We discovered that thymus size, which is generally hypoplastic in *Atm*^*−/−*^ mice, gained weight 7 weeks after transplantation (Fig. 1c). Flow cytometry analysis demonstrated significant increase in CD4 single-positive helper T cells and TCRβ^hi^ thymocytes in transplanted animals, both of which reached levels comparable to wild-type mice (Fig. 1d, e). Furthermore, if *Atm*^*−/−*^ animals normally had reduced numbers of IgG1 positive B cells owing to class switch defects, B cells from 7 weeks transplanted mice showed an increase in IgG1 positive cells, equivalent to wild-type mice (Fig. 1f). We finally observed that male transplanted LK cells indeed reached lymphoid organs in *Atm*^*−/−*^ females as indicated by the presence of the *Sry* male marker and the wild-type *Atm* coding genes (Fig. 1g). Following DNA damage, Atm protein could phosphorylate Atm targets including the transcription factor KAP1, the cell cycle checkpoint proteins Chk1 and Chk2, and the DNA double-strand breaks marker H2AX (Fig. 1h).

We determined that 1 × 10^6^ *Atm*^+*/*+^ LK-enriched cells were sufficient for effective T cell reconstitution in blood, Atm expression and DNA damage response in thymocytes of transplanted *Atm*^*−/−*^ mice (*Atm*^*−/−LKTLow*^), 7 weeks after transplantation (Suppl. Figure 2a–c). To further elucidate the relevance of LK cells in the reconstitution of the immune system of Atm-deficient mice, Lineage negative and c-Kit depleted (Lin^−^c-Kit^−^, LK-) cells were transplanted (Suppl. Figure 3). Notably none of the phenotypes described above were rescued by transplantation of *Atm*^+*/*+^ LK cells, despite they reached the lymphoid organs (Suppl. Figure 3a–h), supporting previous works on the hematopoietic function of c-Kit^+^Sca-1^+^ cells [15].

**Fig. 2:**
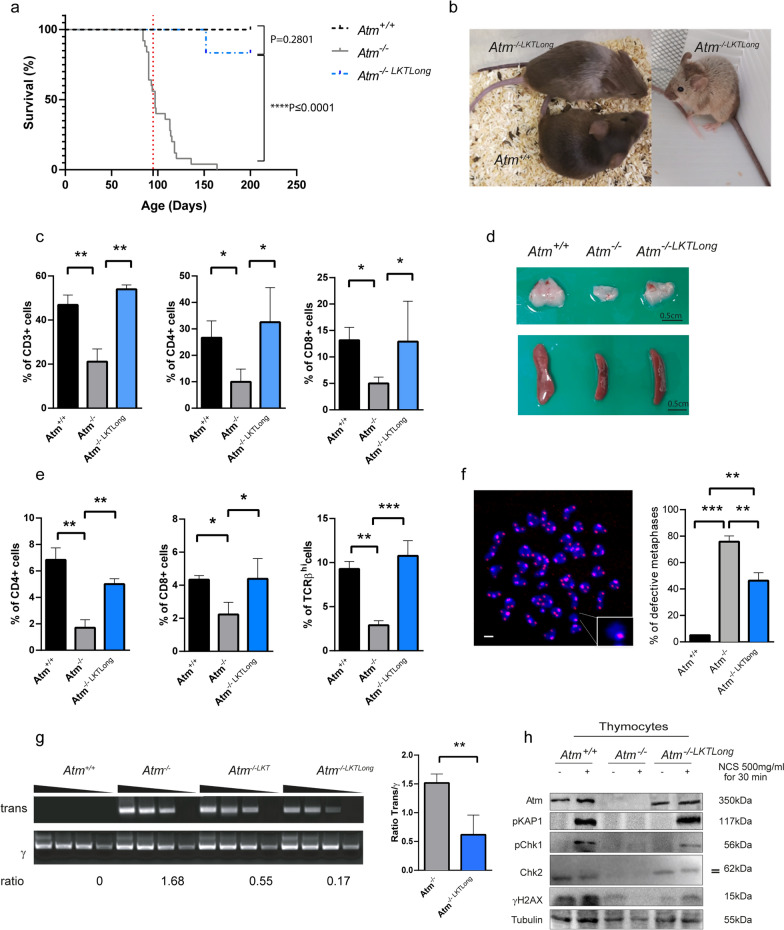
Extended life span and genomic instability prevention after long-term *Atm*^*+*/*+*^ LK transplantation. **a** Kaplan–Meier survival curve Long-rank Mantel-Cox test for *Atm*^+/+^, *Atm*^−/−^ and *Atm*^−/−*LKTLong*^ mice. The red dotted line indicates *Atm*^−/−^ mice median survival (95 days; *Atm*^+/+^ N = 31; *Atm*^−/−^ N = 25; *Atm*^−/−*LKTLong*^ N = 6 **** P ≤ 0.0001). **b** Picture of 7 months old mice. *Atm*^*−/− LKTLong*^ mice present different levels of white/grey fur. **c** Histogram of flow cytometry analysis of blood from 7 months old *Atm*^−/−*LKTLong*^ mice, relative to *Atm*^+/+^ littermate and 2–4 months old *Atm*^−/−^ tumor-free mice. **d** Representative thymus and spleen pictures of 7 months old mice after LKT relative to thymi from *Atm*^+/+^ littermate and 4 months old *Atm*^−/−^ tumor-free mice. **e** Histogram of flow cytometry analysis of isolated thymocytes from 7 months old *Atm*^−/−*LKTLong*^ mice, relative to *Atm*^+/+^ littermate and 2–4 months old *Atm*^−/−^ tumor-free mice. **f** Representative image of FISH assay in B cell metaphases and histogram of defective B cell metaphases. In the inset example of an aberrant chromosome. In red the PNA-bio telomere probe and in blue the DAPI DNA staining. Scale bar = 2 μm. **g** Pictures of PCR for gamma receptor rearrangement (γ) and gamma-beta receptors trans-rearrangement (trans) using as template genomic DNA (500 ng, 100 ng, 10 ng, 1 ng) prepared from thymocytes. The ratio among trans bands and γ is reported for 10 ng dilution PCR product. Examples of short-term (*Atm*^−/−*LKT*^, 7 weeks) and long-term, (*Atm*^−/−*LKTLong*^, 7 months) LK transplantation are reported. Histogram of fold change among trans and γ ratios in thymus of *Atm*^−/−^ and *Atm*^−/−LKTLong^ mice; the weakest bands among different DNA amount were considered. **h** Representative western blot of Atm expression and DNA damage response following NCS treatment in isolated thymocytes of long-term transplanted mice. Phosphorylation of KAP1, Chk1, Chk2 (upper band) and H2AX (γH2AX) indicate DNA damage response. Tubulin was used as loading control. N = 3 mice of each group. * P ≤ 0.05, ** P ≤ 0.01, *** P ≤ 0.001

Long-term study, 6 months post-transplantation, revealed an increase of lifespans and fur whitening in transplanted *Atm*^*−/−*^ mice (*Atm*^*−/− LKTLong*^; Fig. 2a, b), when compared to non-transplanted *Atm*^*−/−*^ mice that usually die at 3–4 months before showing signs of aging in the fur. Further studies will be conducted to address if *Atm*^*−/− LKTLong*^ mice present more aging features. Notably, improvement of the immune systems in blood and thymus with tumor prevention (Fig. 2c–e), decreased chromosome breaks of cultured B cells (Fig. 2f), reduced trans-rearrangement rates in thymocytes (Fig. 2g), and correct DNA damage response in vitro (Fig. 2h) were observed in transplanted mice compared to untreated *Atm*^*−/−*^ animals.

In conclusion, our work shows the potential of *Atm*^+*/*+^ LK cell transplantation to counteract immune deficiency and genomic instability at least till 7 months of age, trough restoration of Non-Homologous End Joining and Homologous recombination repair, respectively, in a mouse model of the A-T disease. Moreover, this study offers the opportunity to investigate gene therapy applications in induced Pluripotent Stem (iPS) cells or HSPCs of A-T patients followed by autologous transplantation.

### Supplementary Information

Supplementary material 1. Figure 1. Enrichment of *Atm*^+/+^ LK progenitor cell population and Flow Cytometry analysis of peripheral blood in non-myeloablative conditioned *Atm*^-/-^ mice. a) Flow cytometry analysis of sequential enrichment of Lin^-^c-Kit^+^ population isolated from total bone marrow of posterior legs of *Atm*^+/+^ mice. The total bone marrow cells (left panels), the cell population recovered after lineage negative selection (middle panels), and the cell population obtained after CD117 beads, either freshly isolated (T0) or cultured for 24 h (T24), (right panels) are shown. The analysis was done with anti APC-Lineage cocktail (upper panels) and with anti PE-CD117 for c-Kit and PE-Cy7 for Sca-1 (lower panels) on gated live cells. Histogram N= 5 experiments; * P≤ 0.05, ** P≤ 0.01. b) Flow cytometry analysis of HSPC population obtained after CD117 beads stained for CD150 stem cell marker and CD48 multipotent progenitor cell marker on freshly isolated (T0) or cultured for 24 h (T24) cells. Histogram N= 3experiments; * P≤ 0.05. c) Representative dot-plot of CD4 and CD8 analysis of *Atm*^-/-^ before conditioning and 4, 7 weeks after conditioning. c) Column charts of helper and cytotoxic T cells (CD4, CD8) in peripheral blood 7 weeks after conditioning. N= 3 mice of each group; ** P≤ 0.01, *** P≤ 0.001. d) Schematic representation of LK transplantation and analysis.

Supplementary material 2. Figure 2. Partial restoration of T cells in *Atm*^-/-^ mice after low *Atm*^+/+^ LK cells transplantation (*Atm*^*−/−LKTLow*^). a) Representative genomic PCR analysis of mouse tissues collected 7 weeks after transplantation of 1x10^6^ *Atm*^+/+^ LK progenitor cells. The *Atm* 200bp band identifies the wild-type sequence of *Atm* gene, whereas the *Atm* 400bp band identifies the knockout sequence of *Atm* gene. N=3 mice for each group. b) Histograms of mean values of CD3 positive and CD4 single positive T cells in blood are shown after 1x10^6^ *Atm*^+/+^ LKT. * P≤ 0.05, ** P≤ 0.01. N=3 mice for each group. c) Western blot analysis of Atm expression and DNA damage response to NCS in isolated thymocytes after 1x10^6^* Atm*^+/+^ LKT. pKAP1 indicates DNA damage response. Tubulin was used as loading control.

Supplementary material 3. Figure 3. *Atm*^-/-^ mice do not rescue immune system after *Atm*^+/+^ LK negative cells transplantation. a) Flow cytometry dot plot of *Atm*^+/+^ LK- population recovered after lineage negative selection and CD117 beads (T0) and cultured for 24 h (T24) before transplantation in *Atm*^-/-^. The analysis was done with anti-CD117 (c-Kit) and anti-Sca-1 antibodies on gated live cells. b) Representative genomic PCR analysis of mouse tissues collected 7 weeks after transplantation of LK positive and LK negative cells. The *Atm* 200bp band identifies the wild-type sequence of *Atm *gene, whereas the *Atm* 400bp band identifies the knockout sequence of *Atm* gene. c) Western blot analysis of Atm expression and DNA damage response in isolated thymocytes. NCS was used as DNA damage inducer. pKAP1 reveals response to DNA damage. Tubulin was used as loading control. d) Dot plots and quantitative analysis of total and helper T cells (CD3, CD4, CD8) in peripheral blood 7 weeks after transplantation. e) Thymus and spleen picture and relative weights. Scale bar 0.5cm. f) Representative flow cytometry panel of CD4 and CD8 thymocytes and relative histogram of mean values of CD4 and CD8 single positive thymocytes. g) Histogram of mean values of TCRβ^hi^ expressing cells. h) Representative flow cytometry dot-plot of B cells class switching cultured with LPS and IL4 for 96 h and relative histogram of mean values of IgG1 expressing cells. N=3 mice of each group; * P≤ 0.05, ** P≤ 0.01, ***P≤ 0.001 and **** P≤ 0.0001.

Supplementary material 4. Supplemental Material and Methods.

## Data Availability

The datasets used and/or analysed during the current study are available from the corresponding author on reasonable request.

## Abbreviations

A-T: Ataxia-Telangiectasia
PID: Primary immunodeficiency
ATM: Ataxia telangiectasia mutated
PI3KK: Phosphatidylinositol-3-kinase related kinase
CSR: Class switch recombination
DSBs: Double-strand breaks
ALL: Acute lymphocytic leukemias
CCL: Chronic T-Cell Leukemias
HLA: Human leukocyte antigen
LK: Lin^−^c-Kit^+^
LK-: Lin^−^c-Kit^−^
PNA: Peptide nucleic acid
NCS: Neocarzinostatin
HSPC: Hematopoietic progenitor cell
HSC: Hematopoietic stem cell
HPC: Hematopoietic progenitor cells
LKT: LK transplantation
