# Supplementary material for: Hematopoietic stem/progenitor cell transplantation recovers immune defects and prevents lymphomas in Atm-deficient mice
Source: Exp Hematol Oncol. 2024 Aug 6;13:81. doi: 10.1186/s40164-024-00544-0 (PMC11302080; doi:10.1186/s40164-024-00544-0)
Supplement: Supplementary file 4 — Supplementary material 4. Supplemental Material and Methods. [file 40164_2024_544_MOESM4_ESM.docx]

**Supplemental Material and Methods**

**Mice.**

The Atm^tm1Awb^ strain^1,2^ in the 129/SVEV/C57BL/6 background was used to generate *Atm^+/+^* and *Atm^-/-^* mice. Mice were kept in an air-conditioned controlled room and fed ad libitum.

## **Hematopoietic progenitor cell enrichment and culture**

*Atm^+/+^* males, 4-6 weeks old, were sacrificed in a CO_2_ chamber. Bone marrow was sterilely isolated from femurs and tibias using the spin isolation method^24^. Bones were placed in 100µl of washing buffer (PBS, pH 7.2, 0.5% bovine serum albumin, 2 mM EDTA) and centrifuged for 10 seconds at 10.000 rpm. Cells were filtered through a 45µm filter (Sarstedt), and red blood cells were lysed with RBC lysis buffer (Sigma-Aldrich) following standard procedures. Lin^-^c-Kit^+^ (LK) cells were obtained by sequential magnetic labeling with Lineage (Direct Lineage Cell Depletion Kit, Miltenyi Biotech) and c-Kit (CD117 Microbeads, Miltenyi Biotech) microbeads according to manufacturer instructions. Before transplantation, enriched LK cells were cultured for 24h in StemSpan serum-free medium (StemCell Technologies) supplemented with the following growth factors (Cell Guidance Systems, UK): murine SCF (100 ng/ml), FLT3L (100 ng/ml), murine THPO (100 ng/ml), and IL-6 (40 ng/ml). c-Kit depleted cells (LK-) were obtained as the flow trough of magnetic beads (CD117 Microbeads, Miltenyi Biotech) and cultured for 24h in StemSpan serum-free medium before transplantation.

##

## **Conditioning and intravenous transplantation of LK progenitor cells.**

*Atm^-/-^* females aged 4 weeks received a non-myeloablative conditioning following a published procedure^3,4^. Irradiation was not used since induces death of *Atm^-/-^* mice^.^ for villi radiosensitivity and consequent diarrhea in four days. Briefly, 0.5 mg anti-CD4 antibody (clone GK1.5, Leinco technologies C1333) 1 mg anti-CD8 antibody (clone YTS 169, Leinco technologies C2850) were intraperitoneal injected 7 days before transplantation and a second dose of each antibody in combination with 200 mg/kg of cyclophosphamide (Sigma-Aldrich) was administered 1 day before transplantation. A total of 1-5×10^6^ cultured LK donor cells or 2-3×10^6^ cultured LK- cells were then injected intravenously into the lateral caudal vein in conditioned female recipients. The use of syngeneic mice and immune deficient *Atm^-/-^* females avoided tissue rejection whereas graft-*versus*-host disease (GvHD), that can affect transplanted mice as humans, was not observed, possibly because of transplantation of immature T and B cells and the use of donor males that do not react against the H-Y antigens^5^. Mice were monitored for the rescue of A-T phenotypes at 4 and 7 weeks or 6 months after transplantation.

##

## **Blood sampling** **and Flow cytometry analysis**

*Atm^+/+^* male donor cells integration on peripheral blood of *Atm^-/-^* females was surveyed at 4- and 7-weeks post-transplantation. Peripheral blood was collected by punching the *vena facialis* into EDTA-coated tubes (Sarstedt) and analyzed by flow cytometry. Thymus and spleen were collected in 4 ml of washing buffer (PBS + 0.05% BSA). Thymocytes were isolated by gentle mincing with round forceps and splenocytes by gentle smashing with the piston syringe. Cells were filtered in 40 µm filters and centrifuged for 8 minutes at 1200 rpm, washed in PBS, counted, and re-suspended for flow cytometry analysis or processed for further analyses. When necessary, cells were treated with RBC lysis buffer (Sigma-Aldrich) to remove red blood cells from the samples. Staining of 1x10^6^ blood, T, B and LK cells was done adding specific antibodies for flow cytometry (Table S1), according to manufacturer’s instructions. Samples were washed in PBS at 300xg for 5 minutes and re-suspended in washing buffer before FACSCanto II (Becton Dickinson, Franklin Lakes, NJ, USA) analysis. At least 30,000 events were collected for each sample. Data analyses were performed by using FlowJo software (Becton Dickinson).

##

## **Class switching, metaphase spreads and in situ fluorescence hybridization**

B cells were isolated from splenocytes with CD43-Ly48 beads (Miltenyi Biotech), plated 3.5x10^6^ cells in a 60 mm dish, and stimulated with LPS (25µg/ml, Sigma-Aldrich) and IL-4 (5ng/ml, Cell Guidance Systems) to induce immunoglobulin class switch. IgG1 staining was carried out 4 days after treatment and analyzed by flow cytometry.

For chromosome instability studies B cells were stimulated for 72h before treatment with 10ug/ml Colcemid (Aurogene) for 3 hours, Fluorescent in situ hybridization (FISH) was performed with a peptide nucleic acid (PNA) probe, following the manufacturer’s protocol (PNA FISH, PNABio, USA). Chromosome images were captured using an Olympus confocal fluorescence microscope and processed using Olympus software (FluoView 4.2c).

## **Genomic PCR** **and V(D)J trans-rearrangements analysis**

Primers for genotyping and to detect the presence of *Atm* and *Sry* genes and *r18S* in the tissues are reported in Table S2. Amplification was performed with MyTaq Mix (Bioline) with initial denaturation of 3 minutes at 95°C, followed by 28 cycles (denaturation 95°C, annealing59°C or 60°C, and extension 72°C for 30 seconds each). Final extension 72°C for 7 minutes (CFX BioRad).

Semiquantitative PCR analysis was performed to target genomic TCR trans-rearrangements in thymocytes. PCR was performed on 500, 100, 10 and 1ng of genomic DNA as previously described^6^ with primers in reported in Table S3.

Nested amplifications were performed with MyTaq Mix (Bioline) with initial denaturation of 2 minutes at 94°C, followed by 25 cycles (denaturation 94°C, annealing 54°C, and extension 72°C, 30 seconds each), final extension 72°C for 7 minutes (CFX BioRad). Bands were quantified with ImageJ software (Fiji 17.13.10).

**Protein extraction and western blot analysis**

Cells isolated for protein analyses were previously treated with or without 500ng/ml Neocarzinostatin (NCS, Sigma-Aldrich) DNA damage inducer for 30 minutes. RIPA buffer (10mM Tris pH 7.4, 50mM NaCl,1mM EDTA, 10mM KCl, 1% NP-40, 0.1% SDS, 0.05% Tween 20) containing protease (Serva) and phosphatase inhibitors (Sigma-Aldrich) was used for protein extraction. Proteins were loaded and resolved on acrylamide/bis-acrylamide (30:1) 7.5% gels (for Atm) or 12%, 15% gels before electrophoretic transfer onto a nitrocellulose membrane (GVS Life Sciences) with semi-dry apparatus (Amersham) overnight at 20 mA for Atm or 1h at 37 mA. Membranes were blocked with 5% non-fat dried milk in TBS-T for 1h at room temperature, then incubated overnight at 4°C with the primary antibodies (Table S4). After washing in TBS-T, membranes were incubated with specific HRP-conjugated secondary antibodies (Rabbit or mouse; 1:5000) for 1h at RT. After washing in TBS-T, blots were developed with Pierce ECL Plus chemiluminescence kit (Thermo Scientific) and signal was detected with ChemiDoc (BioRad). Densitometry analysis was performed by Image J software (Fiji 17.13.10).

## **Statistical Analysis**

All statistical analyses were performed using Prism software, version 8 (GraphPad Software). Data are presented as mean ± SD. One and Two-way ANOVA were used for comparisons between multiple groups. A P value <0.05 was considered statistically significant.

## **References**

1. Pellegrini M, Celeste A, Difilippantonio S, Guo R, Wang W, Feigenbaum L, et al. Autophosphorylation at serine 1987 is dispensable for murine Atm activation in vivo. Nature. 2006;443(7108):222–5.
2. Barlow C, Hirotsune S, Paylor R, Liyanage M, Eckhaus M, Collins F, et al. Atm-Deficient Mice: A Paradigm of Ataxia Telangiectasia. Cell. 1996 Jul;86(1):159–71.
3. Pietzner J, Baer PC, Duecker RP, Merscher MB, Satzger-prodinger C, Bechmann I, et al. Bone marrow transplantation improves the outcome of Atm-deficient mice through the migration of Atm-competent cells. Hum Mol Genet. 2013;22(3):493–507.
4. Bagley J, Cortes ML, Breakefield XO, Iacomini J. Bone marrow transplantation restores immune system function and prevents lymphoma in Atm-deficient mice. Blood. 2004;104(2):572–8.

5. Popli R, Sahaf B, Nakasone H, Lee JYY, Miklos DB. Clinical impact of H-Y alloimmunity.

Immunol Res. 2014; 58(2-3).249-58.

6. Bowen S, Wangsa D, Ried T, Livak F, Hodes RJ. Concurrent V(D)J recombination and DNA

End instability increase interchromosomal trans-rearrangements in ATM-deficient thymocytes.

Nucleic Acids Res. 2013;41(8):4535–48.

Table S1: List of Antibodies for flow cytometry analysis.

| **Cells** | **Antibody** | **Company** |
| --- | --- | --- |
| **Peripheral blood T and B cells;**  **Thymocytes** | CD45-FITC | Becton Dickinson |
|  | CD3- PE-Cy7 | Becton Dickinson |
|  | CD4-FITC | Becton Dickinson |
|  | CD8-PE | Becton Dickinson |
|  | TCRβ-APC | Becton Dickinson |
| **B cells** | CD45R/B220 -FITC | BioLegend |
|  | Streptavidin-PE | BioLegend |
|  | IgG1-biotin | BioLegend |
| **LK cells** | CD34-FITC | Becton Dickinson |
|  | Lineage Antibody Cocktail APC (CD3, CD45R/B220, Ly6C and Ly6G/Gr1, CD11b/Mac1, and TER-119) | Becton Dickinson |
|  | Sca-1-PE-Cy7 | Becton Dickinson |
|  | CD117-PE | Becton Dickinson |
|  | CD150-APC | Immunological Sciences |
|  | CD48-Per-CP-Cy5 | Immunological Sciences |
| **Dead cells** | 7AAD | Becton Dickinson |

Table S2: List of primers used for PCR.

| ***Atm*** | **FW** | GACTTCTGTCAGATGTTGCTGCC |
| --- | --- | --- |
|  | **RV** | CGAATTTGCAGGAGTTGCTGAG |
|  | **NEO** | GGGTGGGATTAGATAAATGCCTG |
| ***18S*** | **FW** | TTTCGGAACTGAGGCCATGATTAAG |
|  | **RV** | AGTTTCAGCTTTGCAACCATACTCC |
| ***Sry*** | **FW** | CAGCCTGCAGTTGCCTCAA |
|  | **RV** | GGTGTGCAGCTCTACTCCAGTCT |

Table S3: List of primers for V(D)J trans-rearrangements analysis.

| **BVA** | **TCRBV5S1a** | TGGTATCAACAGACTCAGGGG |
| --- | --- | --- |
| **BVB** | **TCRBV5S1b** | TTCTCAGTCCAACAGTTTGAT |
| **GVA** | **TCRGV3S1a** | ACCATACACTGGTACCGGCA |
| **GVB** | **TCRGV3S1b** | ACCCCTACCCATATTTTCTTAG |
| **GJA** | **TCRGJ1/2a** | TCATCACTGGAATAAAGCAG |
| **GJB** | **TCRGJ1/2b** | GGTACTTACCGGAGGGAATT |

Supplemental Table S4: List of Antibodies for Western Blot.

| **Western blot Antibodies** | **Company** |
| --- | --- |
| Atm 5C2 1:1000 | Novus |
| Atm D2E2 1:1000 | Cell Signalling |
| pKAP1 (S824) 1:500 | Novus |
| ɣH2AX (S139) 1:1000 | Santa Cruz |
| Chk2 1:500 | Merck Millipore |
| pChk1 (S317) 1:200 | Bethyl |
| β-Tubulin 1:10000 | Invitrogen |
| Vinculin 1:5000 | Sigma-Aldrich |
